# High‐level expression of STING restricts susceptibility to HBV by mediating type III IFN induction

**DOI:** 10.1096/fba.1022

**Published:** 2018-11-05

**Authors:** Hiromichi Dansako, Hirotaka Imai, Youki Ueda, Shinya Satoh, Kunitada Shimotohno, Nobuyuki Kato

**Affiliations:** ^1^ Department of Tumor Virology Okayama University Graduate School of Medicine, Dentistry and Pharmaceutical Sciences Okayama Japan; ^2^ Research Center for Hepatitis and Immunology, National Center for Global Health and Medicine Ichikawa Japan

**Keywords:** hepatitis B virus, hepatocellular carcinoma, host innate immune response, STING, type III interferon

## Abstract

Hepatitis B virus (HBV) is a hepatotropic DNA virus causing hepatic diseases such as chronic hepatitis, liver cirrhosis, and hepatocellular carcinoma. To study HBV, human hepatoma HepG2 cells are currently used as an HBV infectious cell culture model worldwide. HepG2 cells exhibit susceptibility to HBV by exogenously expressing sodium taurocholate cotransporting polypeptide (NTCP). We herein demonstrated that human immortalized hepatocyte NKNT‐3 cells exhibited susceptibility to HBV by exogenously expressing NTCP (NKNT‐3/NTCP cells). By comparing cyclic GMP‐AMP synthetase (cGAS)‐stimulator of interferon genes (STING) signaling pathway in several NKNT‐3/NTCP cell‐derived cell clones, we found that STING was highly expressed in cell clones exhibiting resistance but not susceptibility to HBV. High‐level expression of STING was implicated in HBV‐triggered induction of type III IFN and a pro‐inflammatory cytokine, IL‐6. In contrast, RNAi‐mediated knockdown of STING inhibited type III IFN induction and restored the levels of HBV total transcript in an HBV‐infected cell clone exhibiting resistance to HBV. These results suggest that STING regulates susceptibility to HBV by its expression levels. STING may thus be a novel target for anti‐HBV strategies.

**Abbreviations**
cGAS: cyclic GMP‐AMP synthetase
CsA: cyclosporin A
HBV: hepatitis B virus
HCC: hepatocellular carcinoma
IFN: interferon
ISGs: IFN‐stimulated genes
NTCP: sodium taurocholate cotransporting polypeptide
PAMPs: pathogen‐associated molecular patterns
pgRNA: pregenomic RNA
SNPs: single nucleotide polymorphisms
STING: stimulator of interferon genes

## 1 INTRODUCTION

Hepatitis B virus (HBV) is a hepatotropic virus classified into the Hepadnaviridae family. HBV infection causes chronic hepatitis, liver cirrhosis, and finally hepatocellular carcinoma (HCC).^1^, ^2^ The progression of hepatic diseases is tightly associated with the HBV‐triggered host innate immune response and inflammatory response. To prevent the progression of hepatic diseases, it is important to suppress the HBV‐triggered host innate immune response and inflammatory response.

The cytoplasmic DNA sensor, cyclic GMP‐AMP synthetase (cGAS), is known to recognize viral DNA and cytoplasmic DNA as pathogen‐associated molecular patterns (PAMPs).^3^, ^4^ After the recognition, cGAS produces cyclic GMP‐AMP (cGAMP) and then uses cGAMP to activate a stimulator of interferon genes (STING). STING mediates activation of the transcription factor interferon regulatory factor 3 (IRF‐3) and subsequently the induction of interferon (IFN)‐β (type I IFN),^5^ IFN‐λ1, λ2, and λ3 (type III IFN).^6^ Both type I and type III IFNs stimulate the induction of numerous IFN‐stimulated genes (ISGs) such as ISG15 and ISG56 through the JAK‐STAT signaling pathway.^7^ On the other hand, STING also mediates the induction of pro‐inflammatory cytokines such as IL‐6 and IL‐8 through the NF‐κB signaling pathway.^8^, ^9^ As described here, both cGAS and STING are required for the innate immune response and inflammatory response. We previously reported that cGAS recognized HBV DNA and subsequently triggered an innate immune response in human hepatoma Li23 cells.^10^ However, in that study, we could not examine the HBV‐triggered inflammatory response, since Li23 cells were a human hepatoma cell line. To study HBV‐triggered inflammatory responses, it will be necessary to establish an HBV infectious cell culture model from normal human hepatic cells rather than human hepatoma cells.

Sodium taurocholate cotransporting polypeptide (NTCP) is a functional receptor for HBV.^11^ Human hepatoma HepG2 cells exhibit susceptibility to HBV by exogenously expressing NTCP.^11^ HepG2/NTCP cells (HepG2 cells stably expressing exogenous NTCP) are currently used as an HBV infectious cell culture model for the study of HBV worldwide. However, we previously reported that HepG2 cells exhibited defective expression of endogenous cGAS.^10^ This result suggests that HepG2/NTCP cells cannot be used for the study of endogenous cGAS‐triggered innate immune response and inflammatory response. Our previous study also showed that cGAS was expressed in immortalized human hepatocyte NKNT‐3 cells.^10^ In the present study, we established NKNT‐3 cells exhibiting susceptibility to HBV by the exogenous expression of NTCP. In addition, we obtained several NKNT‐3/NTCP‐derived cell clones exhibiting susceptibility or resistance to HBV. Interestingly, STING was highly expressed in a cell clone exhibiting resistance to HBV. Here, we show that STING is an important host factor that regulates susceptibility to HBV by its expression levels. We also show that NKNT‐3/NTCP cells are a novel HBV infectious cell culture model for the study of HBV‐triggered innate immune responses and inflammatory responses.

## 2 MATERIALS AND METHODS

### 2.1 Cell culture

Human immortalized hepatocyte NKNT‐3 cells, which were kindly provided by N. Kobayashi and M. Namba (Okayama University). Human hepatoma HepG2/NTCP cells were cultured as previously described.^10^ HepG2.2.15 Cont, HepG2.2.15 cGAS/STING, and HepG2.2.15 cGAS GSAA/STING cells were maintained in medium including blasticidin and puromycin as previously described.^10^

### 2.2 Establishment of an NKNT‐3 cell line stably expressing exogenous NTCP and the derivation of its cell clones

NKNT‐3 cells stably expressing exogenous NTCP (designated NKNT‐3/NTCP cells) were established as previously described.^10^ NKNT‐3/NTCP‐derived cell clones were isolated from their parental cells by the limited dilution method. We evaluated HBV susceptibility by HBV/NLuc assay^12^ and, from the several tens of cell clones obtained, selected a cell clone exhibiting susceptibility or resistance to HBV. By repeating the cell cloning and selection process, we obtained cell clones exhibiting the different levels of susceptibility to HBV.

### 2.3 HBV/NLuc assay

HBV/NLuc was prepared as previously reported.^12^ Intracellular NLuc activity was measured at 5, 9, and 13 days after the inoculation of HBV/NLuc. For the measurement of NLuc activity, we used a Nano‐Glo luciferase assay system (Promega, Madison, WI, USA). Data are the means ± SD from three independent experiments.

### 2.4 Western blot analysis

Western blot analysis was performed as previously described.^13^ Anti‐Myc (PL14; Medical & Biological Laboratories, Nagoya, Japan), anti‐ISG15 (H‐150; Santa Cruz Biotechnology, Dallas, TX, USA), anti‐ISG56, anti‐cGAS, anti‐phospho‐STING (Ser366), anti‐STING, anti‐phospho‐NF‐κB p65 (Ser536), anti‐NF‐κB p65 (Cell Signaling Technology, Beverly, MA, USA), and anti‐β‐actin (AC‐15; Sigma‐Aldrich, St. Louis, MO, USA) were used as primary antibodies.

### 2.5 Flow cytometric analysis

Cell surface expression of exogenous NTCP was detected by a flow cytometer as previously reported.^14^ Anti‐Myc (PL14; Medical & Biological Laboratories), and FITC‐conjugated goat anti‐mouse antibody (Jackson ImmunoResearch Laboratories, West Grove, PA, USA) were used as primary and secondary antibody, respectively.

### 2.6 Analysis of HBV RNA

Hepatitis B virus was prepared from the supernatant of HepG2.2.15 cells as previously reported.^10^ Cells were infected with HBV at 10^3^ HBV genome equivalents per cell, unless otherwise described. For the analysis of intracellular HBV RNA after the infection of HBV, we performed quantitative RT‐PCR analysis and Northern blot analysis as previously reported.^10^

### 2.7 Quantitative RT‐PCR analysis

At 5, 9, and 13 days after HBV inoculation or at 6 hours after the transfection of an in vitro‐synthesized ligand, p‐dGdC (Invivogen, San Diego, CA, USA), we performed quantitative RT‐PCR analysis as previously described.^15^ For quantitative RT‐PCR analysis, we used primer sets previously described for ISG56,^16^ IFN‐β,^16^ cGAS,^10^ STING,^10^ IL‐6,^15^ and GAPDH.^15^ We also prepared forward and reverse primer sets for IFN‐λ1 (5’‐ CTGGGAAGGGCTGCCACATT‐3’ (forward) and 5’‐ TTGAGTGACTCTTCCAAGGCG‐3’ (reverse)) and IFN‐λ2/3 (5’‐ CAGCTGCAGGTGAGGGAG‐3’ (forward) and 5’‐CTGGGTCAGTGTCAGCGG‐3’ (reverse)).

### 2.8 RNA interference

The day after mock or HBV infection, we introduced small interfering RNAs (siRNAs) targeting STING or nontargeting siRNAs into NKNT‐3/NTCP #28.3.25.13 cells as previously described.^17^ At 4 days after the introduction of siRNAs, we isolated the total RNA or cell lysate, and subjected it to quantitative RT‐PCR analysis or Western blot analysis, respectively.

### 2.9 Generation of cells stably expressing exogenous STING

To construct pCX4bleo/HA‐STING retroviral vector, we introduced STING (accession no. NM_198282) cDNA containing a full‐length ORF into the pCX4bleo/HA retroviral vector as previously reported.^18^ pCX4bleo/HA‐STING I200N, which causes the conformational disruption of STING,^19^ was also constructed using PCR mutagenesis with primers containing base alterations. These vectors were introduced into NKNT‐3/NTCP #28.3.8 cells by retroviral transfer and then the cells stably expressing exogenous STING or STING I200N were selected by Zeocin (Thermo Fisher Scientific, Carlsbad, CA, USA).

### 2.10 Statistical analysis

Statistical analysis was performed to determine the significance of differences among groups by using Student's *t* test. *P* < 0.05 was considered statistically significant.

## 3 RESULTS

### 3.1 The immortalized human hepatocyte NKNT‐3 cells exhibited susceptibility to HBV via their expression of exogenous NTCP

**Figure 1:**
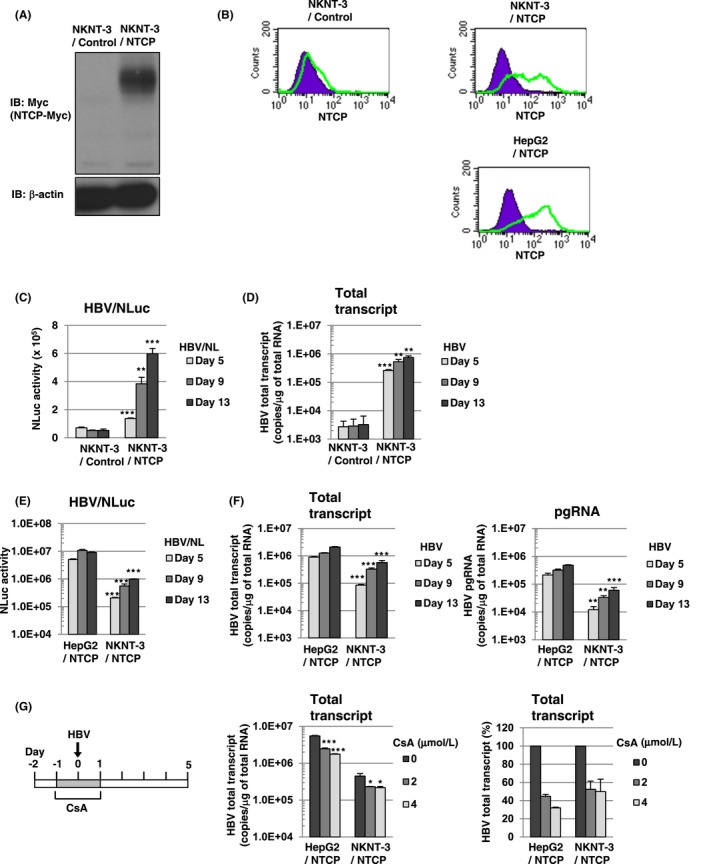
The immortalized human hepatocyte cell line NKNT‐3 exhibited susceptibility to HBV by expressing exogenous NTCP. A, Western blot analysis of exogenous NTCP in NKNT‐3/NTCP cells. Anti‐Myc antibody was used for the detection of NTCP‐Myc in NKNT‐3/NTCP cells. β‐actin was included as a loading control. B, Flow cytometric analysis of the cell surface NTCP in NKNT‐3/Control cells, NKNT‐3/NTCP cells, or HepG2/NTCP cells. Signals of the cell surface NTCP are shown in green. An isotype control was used as a negative control (violet area). C, Comparison of NLuc activity after HBV/NL inoculation between NKNT‐3/Control cells and NKNT‐3/NTCP cells. Intracellular NLuc activity was measured at 5, 9, and 13 d after HBV/NL inoculation. **P < 0.01, ***P < 0.001 versus HBV/NL‐infected NKNT‐3/Control cells. D, Quantitative RT‐PCR analysis of the amount of HBV total transcript in HBV‐infected NKNT‐3/Control cells or NKNT‐3/NTCP cells. The supernatant of HepG2.2.15 cells was used as an HBV inoculum. The amounts of HBV total transcript were measured at 5, 9, and 13 d after HBV inoculation. **P < 0.01, ***P < 0.001 versus HBV‐infected NKNT‐3/Control cells. (E, F) Comparison of the susceptibility to HBV between HepG2/NTCP cells and NKNT‐3/NTCP cells. Intracellular NLuc activity was measured after HBV/NL inoculation. The amounts of HBV total transcript and the pgRNA were measured after HBV inoculation by quantitative RT‐PCR analysis. **P < 0.01, ***P < 0.001 versus HBV/NL‐ or HBV‐infected HepG2/NTCP cells, respectively. G, Functional analysis of NTCP in NKNT‐3/NTCP cells using CsA as an HBV‐entry inhibitor. CsA was administered before and during HBV inoculation. *P < 0.05, ***P < 0.001 versus 0 μmol L^−1^ of CsA‐administered HBV‐infected cells

Since HepG2 cells were a human hepatoma cell line and exhibited defective expression of endogenous cGAS,^10^ we tried to establish HBV infectious cell culture model from immortalized human hepatocyte NKNT‐3 cells, which has been exhibited a nonneoplastic phenotype^20^ and the endogenous expression of cGAS.^10^ HepG2 cells have been reported to exhibit susceptibility to HBV through their expression of exogenous NTCP.^11^ Therefore, to establish NKNT‐3 cells exhibiting susceptibility to HBV, we first prepared NKNT‐3 cells stably expressing exogenous NTCP‐myc (designated NKNT‐3/NTCP cells; Figure 1A). The cell surface expression of NTCP was detected in both NKNT‐3/NTCP cells and HepG2/NTCP cells (HepG2 cells stably expressing exogenous NTCP‐myc), but not in NKNT‐3/Control cells (NKNT‐3 cells stably expressing the control vector) (Figure 1B). By using two kinds of inoculum, HBV/NLuc (genotype C)^12^ and HBV (the supernatant of HBV‐replicating HepG2.2.15 cells, genotype D),^21^ we compared the levels of susceptibility to HBV in NKNT‐3/NTCP cells with that in NKNT‐3/Control cells. After the infection with HBV/NLuc or HBV, both level of NLuc activity and HBV total transcript were increased in NKNT‐3/NTCP cells in a time‐dependent manner, but not in NKNT‐3/Control cells (Figure 1C,D). We next compared the level of susceptibility to HBV in NKNT‐3/NTCP cells with that in HepG2/NTCP cells. The levels of NLuc activity, HBV total transcript, and pregenomic RNA (pgRNA) in HBV/NLuc‐ or HBV‐infected NKNT‐3/NTCP cells were almost 10 times lower than those in HBV/NLuc‐ or HBV‐infected HepG2/NTCP cells (Figure 1E,F). We further examined whether or not the exogenous NTCP was functional in NKNT‐3/NTCP cells. Cyclosporin A (CsA) was previously reported to inhibit HBV entry by targeting NTCP.^22^ When administered before and during HBV inoculation, CsA inhibited the levels of HBV total transcript in HBV‐infected NKNT‐3/NTCP cells as well as in HBV‐infected HepG2/NTCP cells (Figure 1G). These results suggest that NKNT‐3 cells exhibit susceptibility to HBV by exogenously expressing functional NTCP.

### 3.2 The level of susceptibility to HBV in NKNT‐3/NTCP #28.3.8 cells approximated that in HepG2/NTCP cells

**Figure 2:**
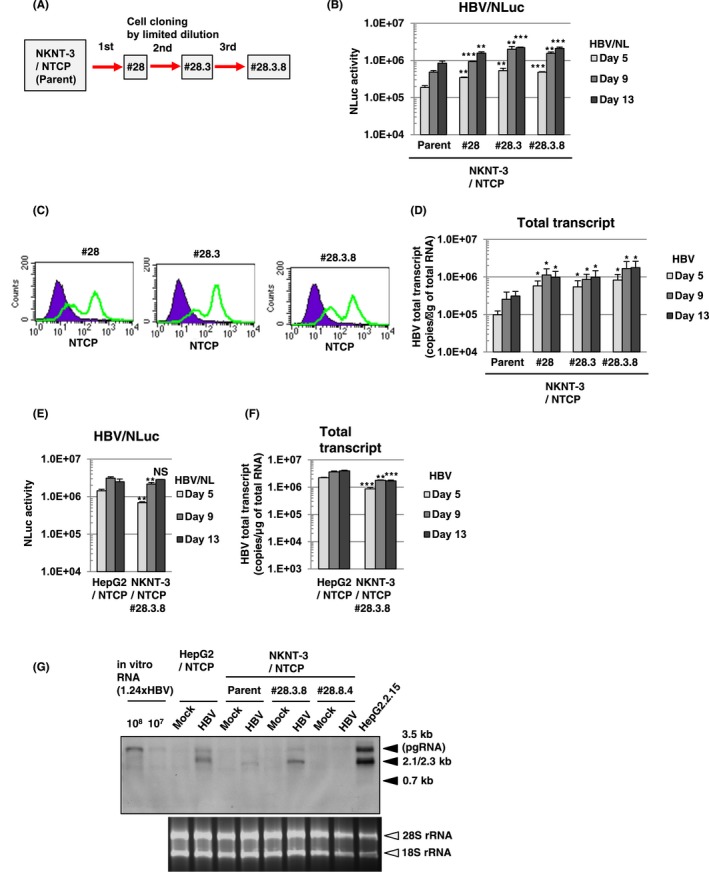
The level of susceptibility to HBV in NKNT‐3/NTCP #28.3.8 cells approximated that in HepG2/NTCP cells. A, Outline of cell cloning by the limited dilution method. NKNT‐3/NTCP #28.3.8 cells were selected by three‐round limited dilution. Red arrows with solid lines show the selection of a cell clone exhibiting higher susceptibility to HBV. B, Comparison of susceptibility to HBV among parent NKNT‐3/NTCP cells and their derived cell clones by using HBV/NL assay. **P < 0.01, ***P < 0.001 versus HBV/NL‐infected parent NKNT‐3/NTCP cells. C, Flow cytometric analysis of the cell surface NTCP in their derived cell clones. Signals of the cell surface NTCP are shown in green. An isotype control was used as a negative control (violet area). D, Comparison of the amounts of HBV total transcript after HBV infection among parent NKNT‐3/NTCP cells and their derived cell clones. The amount of HBV total transcript was measured after HBV infection by quantitative RT‐PCR analysis. *P < 0.05 versus HBV‐infected parent NKNT‐3/NTCP cells. (E, F) Comparison of susceptibility to HBV between HepG2/NTCP cells and NKNT‐3/NTCP #28.3.8 cells. Intracellular NLuc activity or the amounts of HBV total transcript were measured as described in Figure 1E,F. NS; not significant, **P < 0.01, ***P < 0.001 versus HBV/NL‐ or HBV‐infected HepG2/NTCP cells, respectively. G, Comparison of susceptibility to HBV between HepG2/NTCP cells and NKNT‐3/NTCP #28.3.8 cells by Northern blot analysis. Total RNA was isolated from HBV‐infected cells at 13 d after HBV inoculation. 28S rRNA and 18S rRNA were included as a loading control. NKNT‐3/NTCP #28.8.4 is another clone, which has been estimated to exhibit susceptibility to HBV by HBV/NL assay (data not shown)

Since susceptibility to HBV in NKNT‐3/NTCP cells was lower than that in HepG2/NTCP cells (Figure 1E,F), we next tried to select a subcloned cell line exhibiting higher susceptibility to HBV than NKNT‐3/NTCP cells (Figure 2A). During three‐round serial limited dilution, we obtained three distinct cell clones (#28, #28.3, and #28.3.8 cells, respectively; Figure 2A) that met this criterion (Figure 2B). Exogenous NTCP was expressed on the cell surface in all three clones (Figure 2C). Among them, the NKNT‐3/NTCP #28.3.8 cells exhibited the highest levels of HBV total transcript after HBV infection (Figure 2D). Therefore, we next compared the levels of susceptibility to HBV in NKNT‐3/NTCP #28.3.8 cells with those in HepG2/NTCP cells. Upon the infection with HBV/NLuc or HBV, both levels of NLuc activity (Figure 2E) and HBV total transcript (Figure 2F) in NKNT‐3/NTCP #28.3.8 cells approximated those in HepG2/NTCP cells. Consistent with these results, Northern blot analysis also showed that the levels of HBV pgRNA and 2.1/2.3 kb RNA in NKNT‐3/NTCP #28.3.8 cells were roughly the same as those in HepG2/NTCP cells after HBV infection (Figure 2G). These results suggest that NKNT‐3/NTCP #28.3.8 cells are useful as an HBV infectious cell culture model in the manner of HepG2/NTCP cells.

### 3.3 HBV triggered the induction of type III IFNs in NKNT‐3/NTCP #28.3.25.13 cells exhibiting resistance to HBV

**Figure 3:**
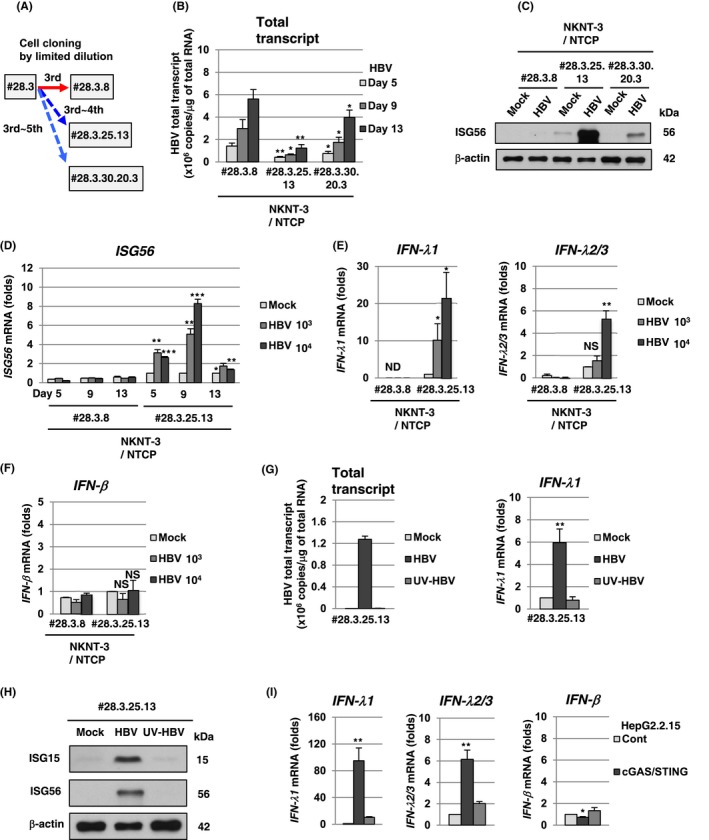
HBV induced type III IFN in NKNT‐3/NTCP #28.3.25.13 cells exhibiting resistance to HBV. A, Outline of cell cloning by the limited dilution method. NKNT‐3/NTCP #28.3.25.13 and #28.3.30.20.3 cells were selected by their distinct serial limited dilution, respectively. Blue arrows with dashed lines show the selection of a cell clone exhibiting resistance to HBV. B, Quantitative RT‐PCR analysis of the amounts of HBV total transcript in HBV‐infected NKNT‐3/NTCP #28.3.8, #28.3.25.13, or #28.3.30.20.3 cells. *P < 0.05, **P < 0.01 versus HBV‐infected NKNT‐3N #28.3.8 cells. C, Western blot analysis of ISG56 in HBV‐infected NKNT‐3/NTCP #28.3.8, #28.3.25.13, or #28.3.30.20.3 cells. Cell lysates were prepared from mock‐ or HBV‐infected cells at 5 d after HBV inoculation. D, Quantitative RT‐PCR analysis of *ISG56* mRNA in HBV‐infected NKNT‐3/NTCP #28.3.8 or #28.3.25.13 cells. Cells were infected with HBV at 10^3^ or 10^4^ HBV genome equivalents per cell, respectively. Each mRNA level was calculated relative to the level in mock‐infected NKNT‐3/NTCP #28.3.25.13 cells, which was set at 1. *P < 0.05, **P < 0.01, ***P < 0.001 versus mock‐infected NKNT‐3N #28.3.25.13 cells. E Quantitative RT‐PCR analysis of *IFN‐λ1* and *IFN‐λ2/3* mRNA in HBV‐infected NKNT‐3/NTCP #28.3.8 or #28.3.25.13 cells. Cells were infected with HBV at 10^3^ or 10^4^ HBV genome equivalents per cell, respectively. Each mRNA level was calculated as described in Figure 3D. ND: not detected. NS: not significant, *P < 0.05, **P < 0.01 versus mock‐infected NKNT‐3/NTCP #28.3.25.13 cells. F, Quantitative RT‐PCR analysis of *IFN‐β* mRNA in HBV‐infected NKNT‐3/NTCP #28.3.8 or #28.3.25.13 cells. Cells were infected with HBV at 10^3^ or 10^4^ HBV genome equivalents per cell, respectively. Each mRNA level was calculated as described in Figure 3D. NS; not significant versus mock‐infected NKNT‐3/NTCP #28.3.25.13 cells. G, (left panel) Quantitative RT‐PCR analysis of the amounts of HBV total transcript in mock‐, HBV‐, or UV‐HBV‐infected NKNT‐3/NTCP #28.3.25.13 cells. (right panels) Quantitative RT‐PCR analysis of *IFN‐λ1* mRNA in mock‐, HBV‐, or UV‐HBV‐infected NKNT‐3/NTCP #28.3.25.13 cells. Each mRNA level was calculated as described in Figure 3D. **P < 0.01 versus mock‐ or UV‐HBV‐infected NKNT‐3/NTCP #28.3.25.13 cells, respectively. H, Western blot analysis of ISG15 and ISG56 in mock‐, HBV‐, or UV‐HBV‐infected NKNT‐3/NTCP #28.3.25.13 cells. The cell lysate was prepared as described in Figure 3C. I, Quantitative RT‐PCR analysis of *IFN‐λ1* ***,*** *IFN‐λ2/3*, and *IFN‐β* mRNA in HepG2.2.15 cGAS/STING cells. Each mRNA level was calculated relative to the level in HepG2.2.15 Cont cells, which was set at 1. *P < 0.05, **P < 0.01 versus HepG2.2.15 Cont cells or HepG2.2.15 cGAS GSAA/STING cells, respectively

During the three‐round limited dilution, we obtained NKNT‐3/NTCP #28.3.8 cells that exhibited higher susceptibility to HBV than the parent NKNT‐3/NTCP cells (Figure 2B,D). On the other hand, during the additional limited dilution (Figure 3A), we unexpectedly obtained a cell clone (#28.3.25.13) exhibiting greater resistance to HBV compared to NKNT‐3/NTCP #28.3.8 cells (Figure 3B). We conjectured that the innate immune response might be induced in cell clones exhibiting resistance to HBV. To examine this possibility, we first compared the HBV‐triggered innate immune responses among cell clones exhibiting susceptibility or resistance to HBV. At 5 days after HBV infection, ISG56 was strongly induced in NKNT‐3/NTCP #28.3.25.13 cells, but not in NKNT‐3/NTCP #28.3.8 cells (Figure 3C). Since HBV‐triggered ISG56 induction in NKNT‐3/NTCP #28.3.25.13 cells was higher than that in #28.3.30.20.3 cells (another cell clone exhibiting resistance to HBV, Figure 3B), we mainly focused the innate immune response to HBV in NKNT‐3/NTCP #28.3.25.13 cells. We first compared the time course of *ISG56* mRNA induction after HBV infection between NKNT‐3/NTCP #28.3.8 and #28.3.25.13 cells (Figure 3D). At 5 or 9 days after HBV infection, *ISG56* mRNA was strongly induced in NKNT‐3/NTCP #28.3.25.13 cells, but not in #28.3.8 cells (Figure 3D). These results suggest that HBV infection induces the innate immune response in cell clone exhibiting resistance but not susceptibility to HBV. We next examined whether type I and/or type III IFN was required for *ISG56* mRNA induction after HBV infection in NKNT‐3/NTCP #28.3.25.13 cells. Interestingly, at 9 days after HBV infection, *IFN‐λ1* and *IFN‐λ2/3* (type III IFN) mRNA, but not *IFN‐β* (type I IFN) mRNA, were induced in NKNT‐3/NTCP #28.3.25.13 cells (Figure 3E,F). In addition, *IFN‐λ1* mRNA (Figure 3G), ISG15 (Figure 3H), and ISG56 (Figure 3H) were induced at 9 days after HBV infection, but not mock or ultraviolet‐inactivated HBV (UV‐HBV) infection, in NKNT‐3/NTCP #28.3.25.13 cells. Consistent with these results, HBV induced *IFN‐λ1* and *IFN‐λ2/3*, but not *IFN‐β* mRNA, in HBV‐replicating HepG2.2.15 cGAS/STING cells stably expressing both exogenous cGAS and STING^10^ (Figure 3I). In addition, the induction levels of *IFN‐λ1* and *IFN‐λ2/3* mRNA in HepG2.2.15 cGAS/STING cells were higher than those in HepG2.2.15 cGAS GSAA/STING cells stably expressing both exogenous cGAS GSAA (the inactive mutant of cGAS) and STING.^10^ These results suggest that HBV induces type III IFN through the cGAS/STING signaling pathway in NKNT‐3/NTCP #28.3.25.13 cells, but not in #28.3.8 cells. These results also suggest that the expression levels of cGAS/STING signaling pathway‐associated host factor(s) are different between NKNT‐3/NTCP #28.3.8 cells and #28.3.25.13 cells.

### 3.4 High‐level expression of STING was implicated in HBV‐triggered type III IFN induction in NKNT‐3/NTCP #28.3.25.13 cells exhibiting resistance to HBV

**Figure 4:**
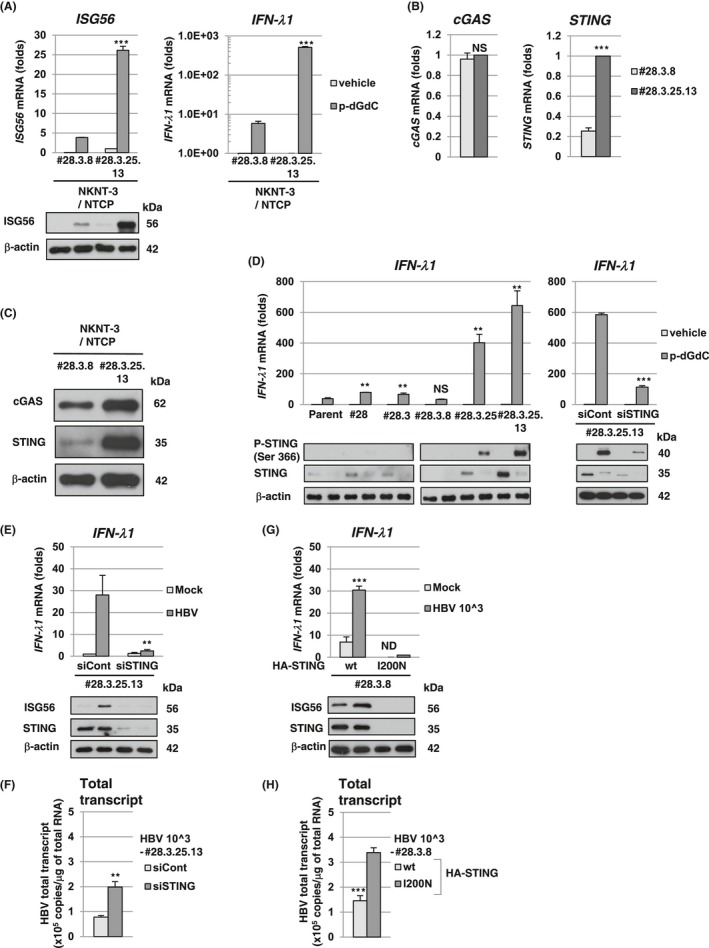
High‐level expression of STING was implicated in HBV‐triggered type III IFN induction in NKNT‐3/NTCP #28.3.25.13 cells. A, (upper panel) Quantitative RT‐PCR analysis of *ISG56* and *IFN‐λ1* mRNA in p‐dGdC‐transfected NKNT‐3/NTCP #28.3.8 or #28.3.25.13 cells. Each mRNA level was calculated relative to the level in vehicle‐transfected NKNT‐3/NTCP #28.3.25.13 cells, which was set at 1. ***P < 0.001 versus p‐dGdC‐transfected NKNT‐3/NTCP #28.3.8 cells. (lower panel) Western blot analysis of ISG56 in p‐dGdC‐transfected NKNT‐3/NTCP #28.3.8 or #28.3.25.13 cells. The cell lysate was prepared as described in Figure 3C. B, Quantitative RT‐PCR analysis of *cGAS* and *STING* mRNA in NKNT‐3/NTCP #28.3.8 or #28.3.25.13 cells. Each mRNA level was calculated relative to the level in NKNT‐3/NTCP #28.3.25.13 cells, which was set at 1. NS; not significant, ***P < 0.001 versus NKNT‐3/NTCP #28.3.8 cells. C, Western blot analysis of cGAS and STING in NKNT‐3/NTCP #28.3.8 or #28.3.25.13 cells. D, (upper‐left panel) Quantitative RT‐PCR analysis of *IFN‐λ1* mRNA in the parent NKNT‐3/NTCP cells and in the several cell clones derived from them after transfection with p‐dGdC. Each mRNA level was calculated relative to the level in NKNT‐3/NTCP #28.3.25.13 cells transfected with vehicle, which was set at 1. NS; not significant, **P < 0.01 versus p‐dGdC‐transfected parent NKNT‐3/NTCP cells. (lower left panel) Western blot analysis of phosphorylated STING at Ser366 in the parent NKNT‐3/NTCP cells and in the several cell clones derived from them after transfection with p‐dGdC. The cell lysate was prepared as described in Figure 3C. (upper‐right panel) Quantitative RT‐PCR analysis of *IFN‐λ1* mRNA in NKNT‐3/NTCP #28.3.25.13 cells transfected with STING‐specific (designated NKNT‐3/NTCP #28.3.25.13 siSTING) or control (designated NKNT‐3/NTCP #28.3.25.13 siCont) siRNA followed by p‐dGdC. Each mRNA level was calculated relative to the level in vehicle‐transfected NKNT‐3/NTCP #28.3.25.13 siCont cells, which was set at 1. ***P < 0.001 versus p‐dGdC‐transfected NKNT‐3/NTCP #28.3.25.13 siCont cells. (lower right panel) Western blot analysis of phosphorylated STING at Ser366 in NKNT‐3/NTCP #28.3.25.13 siSTING cells after transfection with p‐dGdC. The cell lysate was prepared as described in Figure 3C. E, (upper panel) Quantitative RT‐PCR analysis of *IFN‐λ1* mRNA in mock‐ or HBV‐infected NKNT‐3/NTCP #28.3.25.13 siSTING cells or NKNT‐3/NTCP #28.3.25.13 siCont cells. Each mRNA level was calculated relative to the level in mock‐infected NKNT‐3/NTCP #28.3.25.13 siCont cells, which was set at 1. (lower panel) Western blot analysis of ISG56 in HBV‐infected NKNT‐3/NTCP #28.3.25.13 siCont cells or NKNT‐3/NTCP #28.3.25.13 siSTING cells. The cell lysate was prepared as described in Figure 3C. **P < 0.01 versus HBV‐infected NKNT‐3/NTCP #28.3.25.13 siCont cells. F, Quantitative RT‐PCR analysis of the amount of HBV total transcript in HBV‐infected NKNT‐3/NTCP #28.3.25.13 siCont cells or NKNT‐3/NTCP #28.3.25.13 siSTING cells. **P < 0.01 versus HBV‐infected NKNT‐3/NTCP #28.3.25.13 siCont cells. G, (upper panel) Quantitative RT‐PCR analysis of *IFN‐λ1* mRNA in mock‐ or HBV‐infected NKNT‐3/NTCP #28.3.8 cells stably expressing exogenous STING wild type (designated NKNT‐3/NTCP #28.3.8 STING wt) or STING I200N (designated NKNT‐3/NTCP #28.3.8 STING I200N). Each mRNA level was calculated relative to the level in HBV‐infected NKNT‐3/NTCP #28.3.8 STING I200N cells, which was set at 1. ND: not detected. ***P < 0.001 versus HBV‐infected NKNT‐3/NTCP #28.3.8 STING I200N cells. (lower panel) Western blot analysis of ISG56 in HBV‐infected NKNT‐3/NTCP #28.3.8 STING wt cells or NKNT‐3/NTCP #28.3.8 STING I200N cells. The cell lysate was prepared as described in Figure 3C. H, Quantitative RT‐PCR analysis of the amount of HBV total transcript in HBV‐infected NKNT‐3/NTCP #28.3.8 STING wt cells or NKNT‐3/NTCP #28.3.8 STING I200N cells. ***P < 0.001 versus HBV‐infected NKNT‐3/NTCP #28.3.8 STING I200N cells

Since our results suggested that the expression levels of cGAS/STING signaling pathway‐associated host factor(s) were different between NKNT‐3/NTCP #28.3.8 cells and #28.3.25.13 cells, we next compared the levels of p‐dGdC (the synthetic ligand for the cGAS/STING signaling pathway)‐triggered type III IFN induction. We found that the p‐dGdC‐triggered *ISG56* and *IFN‐lambda1* mRNA induction in NKNT‐3/NTCP #28.3.25.13 cells was several times higher than that in NKNT‐3/NTCP #28.3.8 cells (Figure 4A). We next tried to identify the host factor(s) responsible for the higher responsiveness to p‐dGdC in NKNT‐3/NTCP #28.3.25.13 cells. Among cGAS/STING signaling pathway‐associated host factor(s), we found that *STING* mRNA (Figure 4B) and STING protein (Figure 4C) were highly expressed in NKNT‐3/NTCP #28.3.25.13 cells. These results suggest that the high‐level expression of STING enhances p‐dGdC‐triggered type III IFN induction in NKNT‐3/NTCP #28.3.25.13 cells compared to #28.3.8 cells. We further compared the phosphorylation levels of STING among several NKNT‐3/NTCP cell‐derived cell clones. STING was highly phosphorylated in p‐dGdC‐transfected NKNT‐3/NTCP #28.3.25.13 cells, but not in #28.3.8 cells (Figure 4D, lower‐left panel). In addition, STING was also highly phosphorylated in p–dGdC‐treated NKNT‐3/NTCP #28.3.25 cells (the parent cells of #28.3.25.13) but not in the parent cells, or in #28 and #28.3 cells (the common parent cells of #28.3.8, #28.3.25, and #28.3.25.13, respectively). *IFN‐λ1* mRNA was strongly induced in NKNT‐3/NTCP cells highly phosphorylating STING such as NKNT‐3/NTCP #28.3.25 and #28.3.25.13 cells (Figure 4D, upper‐left panel). Consistent with these results, the knockdown of STING reduced *IFN‐λ1* mRNA induction in p‐dGdC‐transfected NKNT‐3/NTCP #28.3.25.13 cells (Figure 4D, upper‐right panel). These results suggest that STING regulate p‐dGdC‐triggered type III IFN induction by its expression level in NKNT‐3/NTCP cells.

We next examined whether high‐level expression of STING was required for HBV‐triggered type III IFN induction in NKNT‐3/NTCP #28.3.25.13 cells. We found that knockdown of STING decreased the induction of *IFN‐λ1* mRNA (Figure 4E, upper panel) and subsequently ISG56 (Figure 4E, lower panel) in HBV‐infected NKNT‐3/NTCP #28.3.25.13 cells. The knockdown of STING also increased the amounts of HBV total transcript in HBV‐infected NKNT‐3/NTCP #28.3.25.13 cells (Figure 4F). On the other hand, the stable expression of exogenous STING, but not STING I200N which causes the conformational disruption,^19^ increased the induction of *IFN‐λ1* mRNA (Figure 4G, upper panel) and subsequently ISG56 (Figure 4G, lower panel) in HBV‐infected NKNT‐3/NTCP #28.3.8 cells. The stable expression of exogenous STING also decreased the amounts of HBV total transcript in HBV‐infected NKNT‐3/NTCP #28.3.8 cells (Figure 4H). These results suggest that high‐level expression of STING is implicated in HBV‐triggered type III IFN induction in NKNT‐3/NTCP #28.3.25.13 cells.

### 3.5 High‐level expression of STING was required for the HBV‐triggered inflammatory response in NKNT‐3/NTCP #28.3.25.13 cells

**Figure 5:**
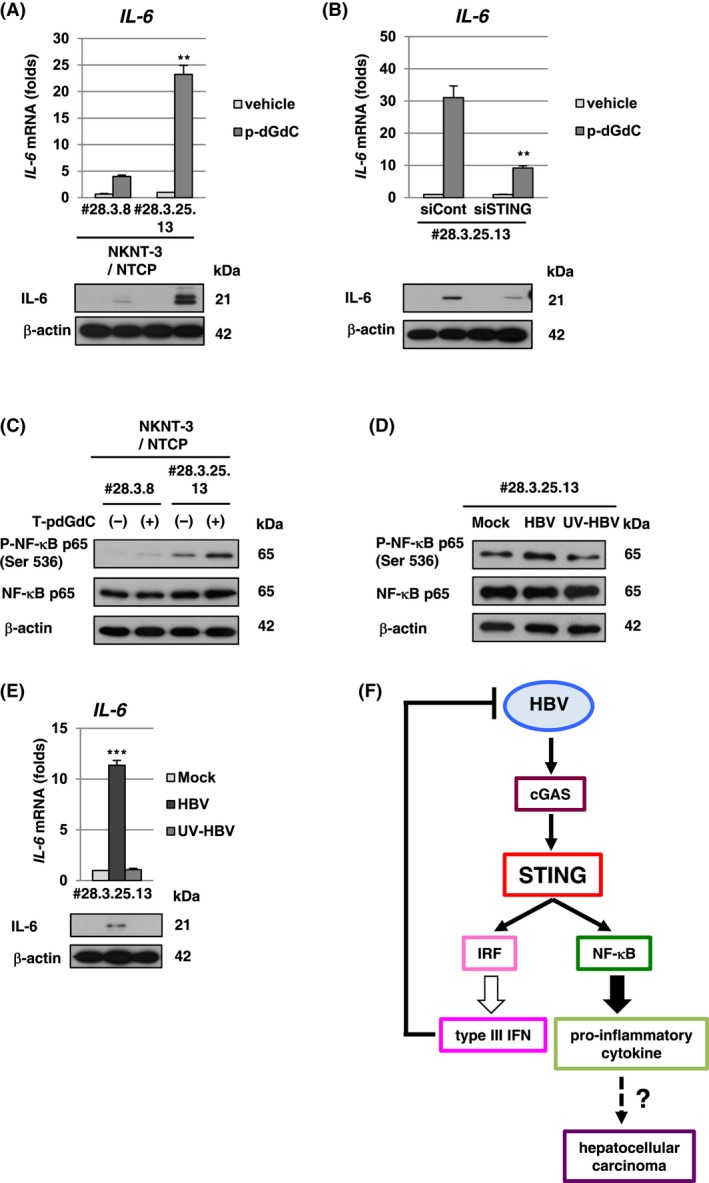
High‐level expression of STING was required for HBV‐triggered inflammatory response in NKNT‐3/NTCP #28.3.25.13 cells. A, (upper panel) Quantitative RT‐PCR analysis of *IL‐6* mRNA in p‐dGdC‐transfected NKNT‐3/NTCP #28.3.8 or #28.3.25.13 cells. Each mRNA level was calculated relative to the level in vehicle‐transfected NKNT‐3/NTCP #28.3.25.13 cells, which was set at 1. **P < 0.01 versus p‐dGdC‐transfected NKNT‐3/NTCP #28.3.8 cells. (lower panel) Western blot analysis of IL‐6 in p‐dGdC‐transfected NKNT‐3/NTCP #28.3.8 or #28.3.25.13 cells. The cell lysate was prepared as described in Figure 3C. B, (upper panel) Quantitative RT‐PCR analysis of *IL‐6* mRNA in p‐dGdC‐transfected NKNT‐3/NTCP #28.3.25.13 siCont cells or NKNT‐3/NTCP #28.3.25.13 siSTING cells. Each mRNA level was calculated relative to the level in vehicle‐transfected NKNT‐3/NTCP #28.3.25.13 siCont cells, which was set at 1. **P < 0.01 versus p‐dGdC‐transfected NKNT‐3/NTCP #28.3.25.13 siCont cells. (lower panel) Western blot analysis of IL‐6 in p‐dGdC‐transfected NKNT‐3/NTCP #28.3.25.13 siCont cells or NKNT‐3/NTCP #28.3.25.13 siSTING cells. The cell lysate was prepared as described in Figure 3C. C, Western blot analysis of phosphorylated NF‐κB p65 at Ser536 in p‐dGdC‐transfected NKNT‐3/NTCP #28.3.8 or #28.3.25.13 cells. D, Western blot analysis of phosphorylated NF‐κB p65 at Ser536 in mock‐, HBV‐, or UV‐HBV‐infected NKNT‐3/NTCP #28.3.25.13 cells. E (upper panel) Quantitative RT‐PCR analysis of *IL‐6* mRNA in mock‐, HBV‐, or UV‐HBV‐infected NKNT‐3/NTCP #28.3.25.13 cells. Each mRNA level was calculated as described in Figure 3D. ***P < 0.001 versus mock‐ or UV‐HBV‐infected NKNT‐3/NTCP #28.3.25.13 cells, respectively. (lower panel) Western blot analysis of IL‐6 in mock‐, HBV‐, or UV‐HBV‐infected NKNT‐3/NTCP #28.3.25.13 cells. F, Proposed model of the HBV‐triggered host innate immune response and inflammatory response through STING

Since high‐level expression of STING‐mediated HBV‐triggered type III IFN induction in NKNT‐3/NTCP #28.3.25.13 cells (Figure 4D,E), we next examined whether high‐level expression of STING was implicated in the induction of not only type III IFN but also pro‐inflammatory cytokine including IL‐6 through the NF‐κB signaling pathway. *IL‐6* mRNA induction in p‐dGdC‐transfected NKNT‐3/NTCP #28.3.25.13 cells was higher than that in p‐dGdC‐transfected NKNT‐3/NTCP #28.3.8 cells (Figure 5A). In addition, the knockdown of STING reduced *IL‐6* mRNA induction in p‐dGdC‐transfected NKNT‐3/NTCP #28.3.25.13 cells (Figure 5B). Since the phosphorylation of NF‐κB p65 at Ser536 was required for the activation of noncanonical NF‐κB signaling pathway,^23^ we next compared the phosphorylation of NF‐κB p65 at Ser536 between p‐dGdC‐transfected NKNT‐3/NTCP #28.3.8 cells and #28.3.25.13 cells. Our results indicated that NF‐κB p65 was phosphorylated at Ser536 in p‐dGdC‐treated NKNT‐3/NTCP #28.3.25.13 cells, but not #28.3.8 cells (Figure 5C). These results suggest that high‐level expression of STING enhances p‐dGdC‐triggered *IL‐6* mRNA induction through the noncanonical NF‐κB signaling pathway in NKNT‐3/NTCP #28.3.25.13 cells. We next examined whether HBV infection also triggered *IL‐6* mRNA induction through the noncanonical NF‐κB signaling pathway in NKNT‐3/NTCP #28.3.25.13 cells. Interestingly, HBV infection, but not mock or UV‐HBV infection, triggered the phosphorylation of NF‐κB p65 at Ser536 (Figure 5D) and subsequently induced *IL‐6* mRNA (Figure 5E) in NKNT‐3/NTCP #28.3.25.13 cells. These results suggest that high‐level expression of STING is implicated in HBV‐triggered pro‐inflammatory cytokine induction through the noncanonical NF‐κB signaling pathway in NKNT‐3/NTCP #28.3.25.13 cells. NKNT‐3/NTCP #28.3.25.13 cells are a useful tool for studying hepatic carcinogenesis caused by the HBV‐triggered inflammatory response through the NF‐κB signaling pathway.

## 4 DISCUSSION

Cytoplasmic DNA or RNA sensors trigger the innate immune responses and the inflammatory responses by recognizing viral PAMPs. We previously reported that one of the cytoplasmic DNA sensors, cGAS, recognized HBV DNA as viral PAMPs and subsequently induced the innate immune response through its adaptor protein, STING.^10^ In the present study, we found that the immortalized human hepatocyte NKNT‐3 cells exhibited HBV susceptibility by stably expressing the exogenous NTCP (Figure 1C,D). Cells of one of the NKNT‐3/NTCP cell‐derived clones, NKNT‐3/NTCP #28.3.25.13, highly expressed STING and exhibited resistance to HBV through STING‐mediated type III IFN induction (Figure 4C,E,F). Interestingly, STING was highly phosphorylated in p–dGdC‐transfected NKNT‐3/NTCP #28.3.25.13 cells, but not in the parent, #28, #28.3, or #28.3.8 cells (Figure 4D). However, it is uncertain why the expression and phosphorylation levels of STING differed among the NKNT‐3/NTCP cell‐derived cell clones. In humans, several single nucleotide polymorphisms (SNPs) of STING have been discovered.^24^ SNPs of STING have been shown to cause autoinflammatory diseases such as STING‐associated vasculopathy with onset in infancy^25^ and familial chilblain lupus.^26^ These SNPs are implicated in the dysregulation of host innate immune responses and inflammatory responses through a loss‐of‐function mutation or a gain‐of‐function mutation of STING. Further analysis is needed to identify the gain‐of‐function mutation(s) in STING in NKNT‐3/NTCP #28.3.25.13 cells.

In the present study, we showed that HBV infection induced type III IFN, but not IFN‐β (type I IFN), through a STING‐mediating signaling pathway in NKNT‐3/NTCP #28.3.25.13 cells (Figure 5F). Sato et al previously reported that a cytoplasmic RNA sensor, RIG‐I, recognized HBV pgRNA and subsequently induced type III but not type I IFN through its adaptor protein, IPS‐1, in human primary hepatocytes.^27^ These results suggest that HBV suppresses the induction of type I IFN but not type III IFN. One of the HBV proteins, HBV polymerase, suppressed STING‐mediated IFN‐β induction by disrupting K63‐linked ubiquitination of STING.^28^ Another study also reported that HBx bound IPS‐1 and suppressed the activation of IFN‐β.^29^ However, in these studies, it was unclear whether HBV suppressed the induction of type III IFN through these HBV proteins. Our results showed that HBV transiently induced *ISG56* mRNA induction at 5 and 9 days, but not at 13 days, after HBV infection in NKNT‐3/NTCP #28.3.25.13 cells (Figure 3D). This result suggests that HBV possesses two opposite functions to simultaneously trigger or suppress the induction of type III IFN. Further analysis is needed to examine whether or not HBV suppresses the induction of type III IFN.

We also showed that HBV infection induced a pro‐inflammatory cytokine, IL‐6, through the noncanonical NF‐κB signaling pathway in NKNT‐3/NTCP #28.3.25.13 cells (Figure 5F). STING also mediates host inflammatory responses by triggering its downstream NF‐κB signaling pathway.^8^, ^9^ A STING‐triggered host inflammatory response has been reported to be associated with hepatic diseases.^30^, ^31^ In nonalcoholic fatty liver disease, STING promotes hepatocyte injury by inducing inflammation.^30^ In addition, STING mediates liver injury and fibrosis in mice administered CCl_4_ (a chemical inducer of hepatocyte death).^31^ Moreover, based on the results of several previous studies, STING is also thought to play an important role in tumor development.^32^ Interestingly, STING may exert two opposite effects (tumor‐suppressing and tumor‐promoting effects) on tumor development under different situations. For example, in breast cancer, STING and its downstream signaling may suppress the tumor or the cancer metastasis.^33^, ^34^ In contrast, STING is also required for cell survival and regrowth in breast cancer.^35^, ^36^ However, the results of the present study do not clarify whether the HBV‐triggered NF‐κB signaling pathway causes liver diseases and tumor development. Further analysis will also be needed to examine how HBV causes liver diseases and finally HCC through a STING‐mediated NF‐κB signaling pathway.

In the present study, we established a novel HBV infectious cell culture model by using NKNT‐3 cells. Since NKNT‐3 cells exhibit a nonneoplastic phenotype,^20^ our HBV infectious cell culture model is expected to be a useful tool for the study of hepatic carcinogenesis caused by HBV‐triggered innate immune responses and inflammatory responses.

## CONFLICT OF INTEREST

The authors declare that they have no conflict of interest.

## AUTHOR CONTRIBUTIONS

H. Dansako and N. Kato designed research; H. Dansako performed most of research; H. Imai contributed the preparation of pCX4bleo HA‐STING I200N; N. Kato performed the cell cloning by the limited dilution method; K. Shimotohno contributed HBV/NLuc assay; H. Dansako, H. Imai, Y. Ueda, S. Satoh and N. Kato analyzed data; H. Dansako wrote the paper; and all authors reviewed the manuscript.
